# Investigation of Nonlinear Output-Input Microwave Power of DMSO-Ethanol Mixture by Molecular Dynamics Simulation

**DOI:** 10.1038/s41598-018-21846-4

**Published:** 2018-05-08

**Authors:** Min Zhou, Ke Cheng, Haoran Sun, Guozhu Jia

**Affiliations:** 1 College of Physical and Electronics Engineering, Sichuan Normal University, Chengdu, 610101 China; 2 College of Optoelectronic Technology, Chengdu University of Information Technology, Chengdu, 610103 China

## Abstract

The nonlinear response of output-input microwave power for DMSO-ethanol mixture, which was exhibited as the direct evidence of non-thermal effect in experiment, was investigated by molecular dynamics simulation. Effects of microwave field on the mixture were evaluated from the alteration in structure, transport, hydrogen bonding dynamics and intermolecular interaction energy. Increasing the strength of the microwave field did not lead to any markedly conformational change, but decrease the diffusion coefficient. Prolonged hydrogen bonding lifetimes, which caused by the redistribution of microwave energy, was also detected. Distinct threshold effect was observed, which was consistent with the behavior in the experiment.

## Introduction

Microwave irradiation as non-conventional energy source plays a leveraged role in chemical transformations (e.g., organic synthesis^1–5^, polymer chemistry^6,7^, materials science^8,9^, nanotechnology^10^ and biochemical processes^11–14^). It offers considerable advantages for accelerating chemical reaction, including shortening reaction time, enhancing product yield and purity^15–17^, comparing with conventional heating methods. However, there is an ongoing controversy over the nature of microwave-assisted acceleration^18–22^. Most chemists today will agree that the observed enhancements in microwave heated reactions are the consequence of pure thermal/kinetic effects^15,19,23^. Because they believe that the absorption of microwave photons is far too low to cause any chemical bond breaking, and that therefore microwaves could not “induce” molecules to undergo chemical reactions^24,25^. Nevertheless, there are reports which also demonstrate the existence of “specific” on “non-thermal” microwave effects^22,26,27^.

**Figure 1:**
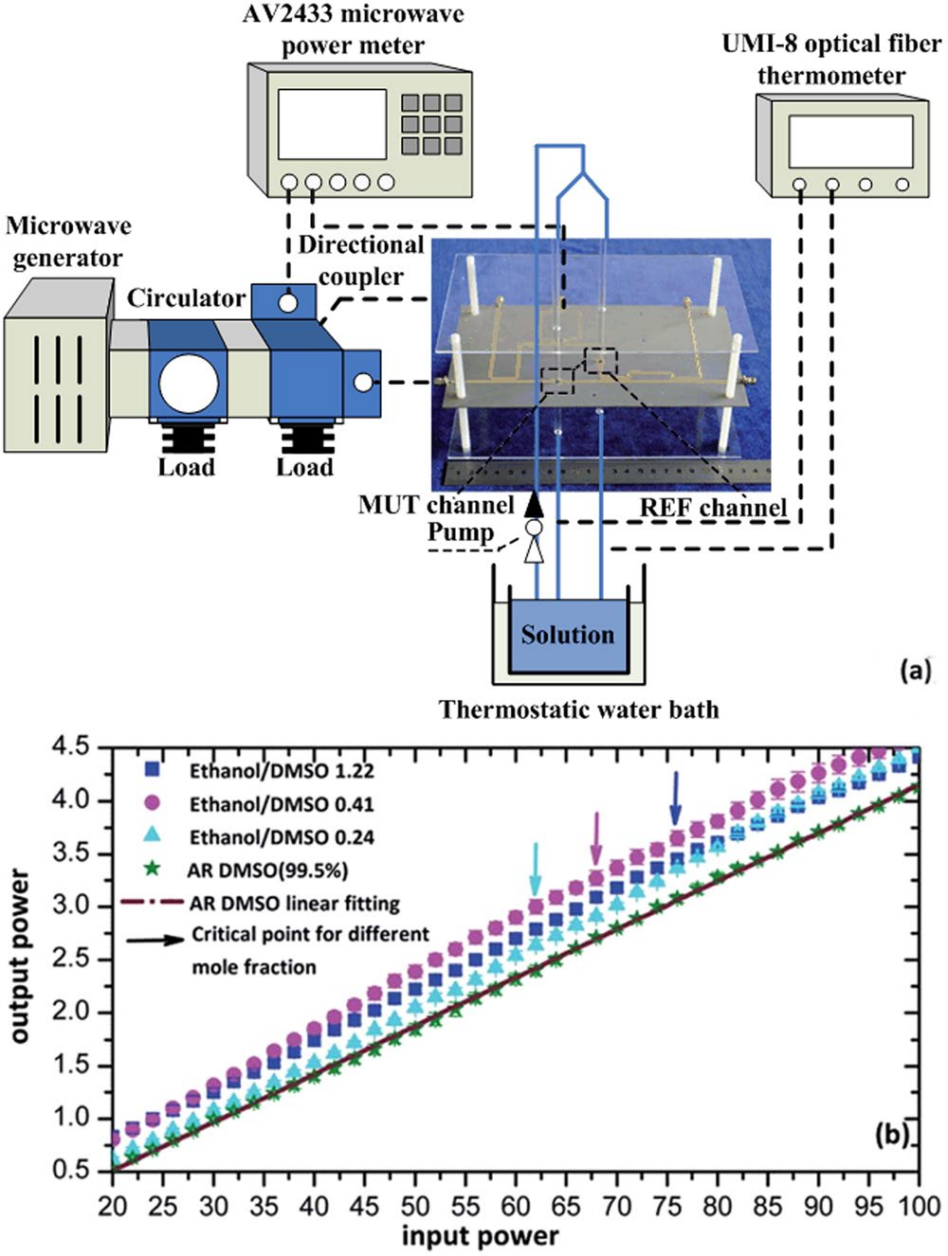
(**a**) Diagram of the experimental system. The combination of microwave generator, circulator, directional coupler and load is utilized to generate a stable and minimum amount of microwave power with a fixed frequency of 2.45 GHz. Two quartz glass pipelines are continuously pumped the equal part of the measured solution, as the material under the test (MUT) and reference (REF) respectively. The input and output microwave power of MUT are detected by microwave power meter, and the dielectric property changes of solution in flowing channel are monitored by a high-sensitivity radio frequency sensor displayed at the center of the diagram. In order to exclude the microwave heating-induced temperature effects, the thermostat and UMI-8 optical fiber thermometer are employed to precisely control the temperature of measured solution at 300 K (±0.05 °C). (**b**) The relationship of the output versus input microwave power for DMSO-ethanol mixtures at different mole fractions and pure DMSO (99.5%) solution. The “critical point” is tagged as the point where the nonlinear characteristic of the curve first emerged and corresponds to the field amplitude of 1.0 × 10^5^ V/m. Reproduced with permission^46^. Copyright 2015, The Royal Society of Chemistry.

Microwave non-thermal effects have been postulated to result from a direct, often stabilizing interaction of the electromagnetic field with specific molecules, intermediates, or even transition states in the reaction medium that is not related to a macroscopic temperature effect^21^. It has been suggested that understanding the non-thermal effect of microwave field with the systems under consideration is of great importance to the development of novel separation technologies^28,29^, selective heating, heterogeneous catalysis^30,31^, and in solid phase organic synthesis (SPOS)^32–35^. Over the past decades, a number of experiential techniques^21,36–41^ and theoretical methods^42–45^ have been carried out to illustrate the non-thermal microwave effects. Investigating the variation of dielectric property caused by external electric fields (and therefore of the microwave power) is an effective way to understand the microwave non-thermal mechanism^46^. Based on our previously experimental system (see Fig. 1a), dielectric property changes of Dimethyl sulfoxide (DMSO) -ethanol (EOH) mixtures under microwave field are investigated, the non-thermal effects are demonstrated owing to the non-linearity ratio between output and input microwave power^46^. Interestingly, we found that only the combination of DMSO-primary alcohol mixtures remarkably presences this effect among the numerous binary mixtures.

DMSO is extensively used in organic chemistry, industry, cryoprotection and biology^47–50^. It consists of a highly polar S=O group, which interacts easily with water forming strong hydrogen bond, and two hydrophobic -CH3 groups^51,52^. Ethanol is widely applied as a chemical reagent, solvent, paint stripper, fuel, and a component in alcoholic beverages^53^. The hydroxyl group (-OH) is tending to attract partially positive hydrogen atoms of another ethanol molecules to form winding hydrogen chain structure in liquid ethanol^54^. Both of them are commonly used in microwave heating as polar solvents. Several works have demonstrated that DMSO-ethanol mixture exhibits properties deviating from ideal due to the intrinsical variation of hydrogen bonding interaction^55–58^. In the case of experiment, we have deduced that the nonlinear behaviors of the DMSO-ethanol mixture are related to the alteration of hydrogen bonds, which caused by the application of microwave. However, for the restrictions in experimental conditions, we only measure the ratio of output-input power. There is not any direct experimental method to observe the concomitant effect of the microwave field at the molecular level.

Molecular dynamics (MD) simulation proves an viable and potentially valuable way in studying the effects of external electric field in molecular systems, it has been utilized to provide an significant insight for understanding the microwave non-thermal effects on water^59–61^, hydrates^62^, metal oxides^63^, zeolites^64,65^ and polystyrene solutions^28^. Thus, in order to further interpret the microwave non-thermal effect in our experiment, a series of molecular dynamics simulations were performed to investigate the structure, transport property, hydrogen bond dynamics and intermolecular interaction energy of DMSO-ethanol mixture under the microwave field. What’s more, mixture with ethanol mole fraction ${X}_{EOH}=0.41$ is chosen as the subjects of the MD simulation due to its excellent performances in experiment^46^. The results and discussion of previous experiment are briefly explained in the part of experimental results.

## Experimental Results

Figure 1 depicts the experimental system and the ratio of output versus input microwave power with different mole fraction DMSO-ethanol mixtures and pure DMSO. The interaction between microwave and substances mainly embodies in microwave absorption and reflection, which strongly correlate with the medium’s dielectric property^58^. Nonlinear output-input microwave power appears after a threshold (“critical point”) input power suggests the redistribution of microwave energy and dielectric property changes in DMSO-ethanol mixtures, which are viewed as evidence of non-thermal microwave effect. On the contrary, the curves representing the sole DMSO liquid and the other binary mixtures (no plot) are maintain linear.

## Results and Discussion

### Structure

**Figure 2:**
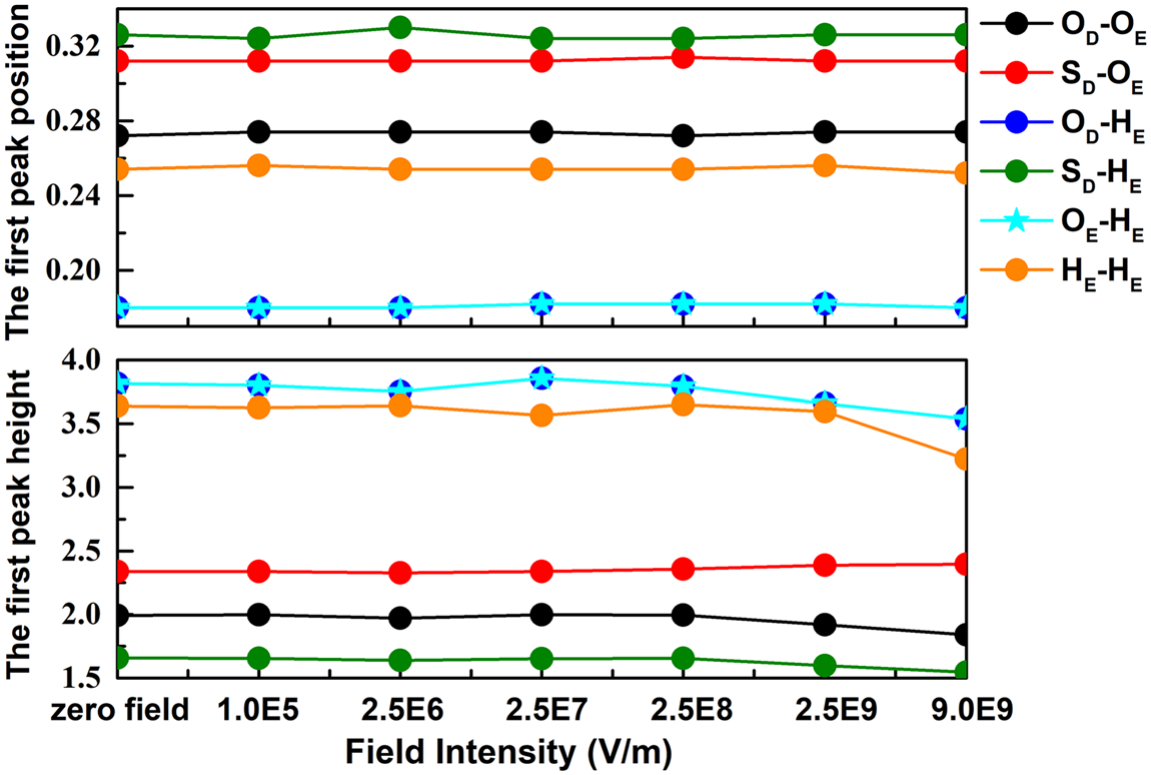
The position and the height of the first peaks of radial distribution functions for pairs in DMSO-ethanol mixture as a function of external field intensity: (**a**) O_D_-O_E_; (**b**) S_D_-O_E_; (**c**) O_D_-H_E_; (**d**) S_D_-H_E_; (**e**) O_E_-H_E_ and (**f**) H_E_-H_E_. Where H_E_ is the hydrogen atom of the hydroxyl group of ethanol molecules.

**Figure 3:**
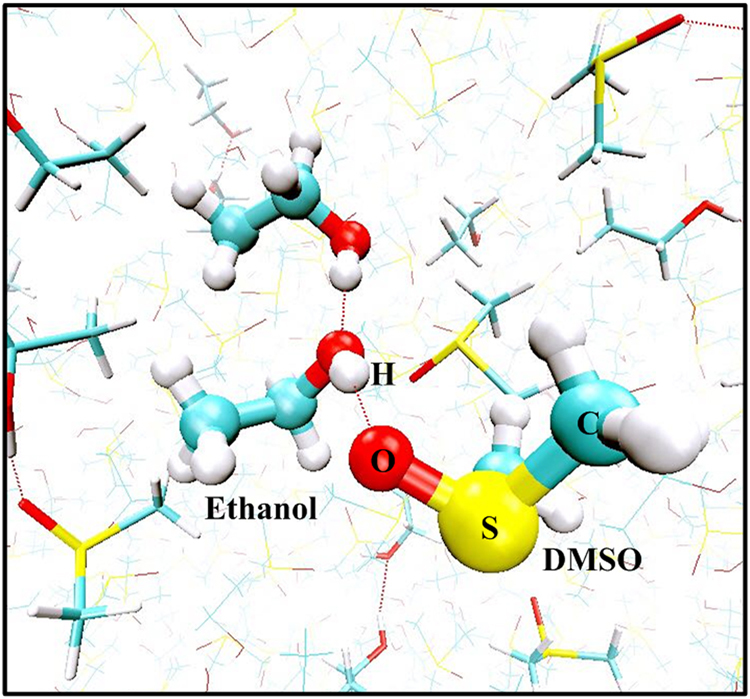
Snapshot of part of the simulation box corresponding to ${E}_{{\rm{\max }}}=zero$ showing DMSO-ethanol and ethanol-ethanol hydrogen bonds. For better visualization, one of the DMSO molecules along with the nearest hydrogen-bonded ethanol molecule is shown in CPK representation; other DMSO and ethanol molecules are represented in licorice and line styles, respectively. The picture was generated with the help of VMD package.

In order to detect the conformational changes of DMSO-ethanol mixture under the microwave field, the first peak position and the height of radial distribution functions (RDFs), *g*_*αβ*_(*r*), involving the O_D_-O_E_, S_D_-O_E_, O_D_-H_E_, S_D_-H_E_, O_E_-H_E_ and H_E_-H_E_ pairs of sites are depicted in Fig. 2. The results are reported in Fig. 2 for the position of first RDF peak maintain immobile with application of the microwave field. The height of the first peak for O_D_-H_E_, O_E_-H_E_ and H_E_-H_E_ pairs slightly varies for ${E}_{{\rm{\max }}} > 2.5\times {10}^{6}$ V/m and eventually decrease with the increased electric field strength. It is attributed to the molecular rotation following the external electric field^60^. Noting that the first peaks for O_D_-H_E_ and O_E_-H_E_ pairs are exactly the same in height and position, this distribution is conducive to creating mutually reinforce hydrogen bonds and enhance the stability or the structure. Indeed, liquid ethanol tends to form winding chain-like hydrogen bonds^54^, when DMSO solvent is dissolved, they are having a tendency to form dimers or timers in mixtures owing to the hydrogen bond interaction (Fig. 3)^57^. Thus, these results indicate that the fluid structuring remains unchanged with applying a sufficient e/m field.

### Transport property

**Figure 4:**
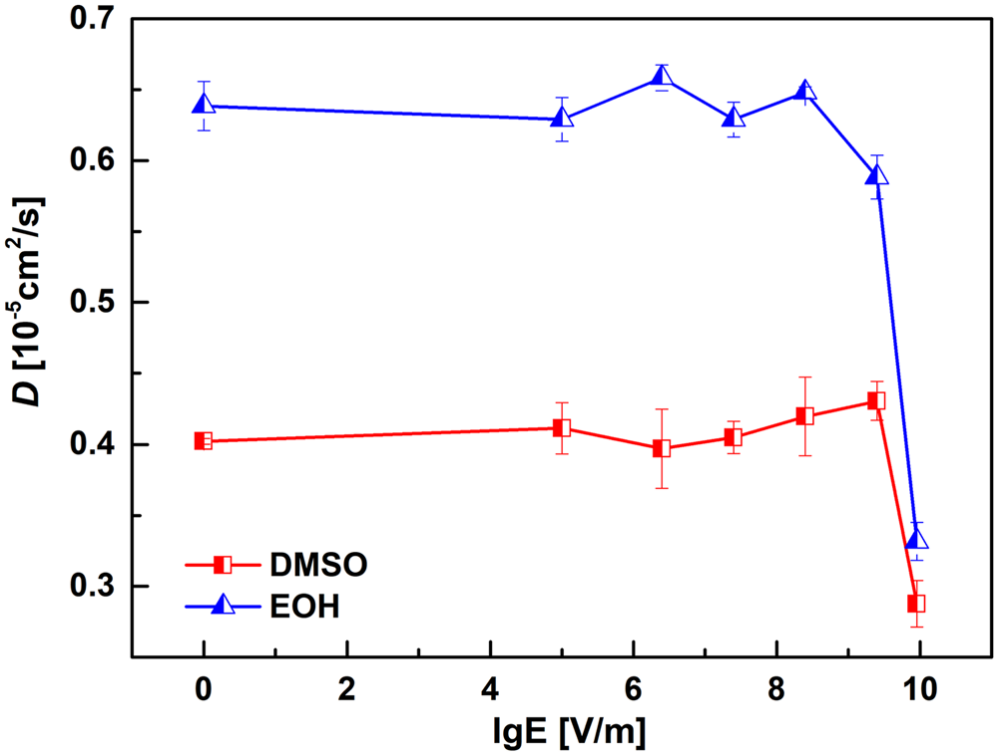
Self-diffusion cofficients as the function of the applied field intensity for DMSO and ethanol.

Molecular transport has been estimated via the self-diffusion coefficients, which are calculated by Einstein’s equation (Eq. 1) with the appropriate slope of MSDs^66^.

1 $$D=\frac{1}{6}\mathop{\mathrm{lim}}\limits_{t\to \infty }\frac{d}{dt}\langle \sum _{i}^{N}{[{r}_{i}(t)-{r}_{i}(0)]}^{2}\rangle $$

where *r*_*i*_(*t*) and *r*_*i*_(0) are position vectors of the center mass of a water molecule *i* at time *t* and 0, respectively. Angular brackets $\langle \mathrm{...}\rangle$ represent an ensemble average. The calculated results are depicted in Fig. 4, the self-diffusion coefficients both of the DMSO and the ethanol fluctuate in a small amplitude at low intensities ($1.0\times {10}^{5} < {E}_{{\rm{\max }}} < 2.5\times {10}^{9}$ V/m) whereas sharply decline at ${E}_{{\rm{\max }}}=9.0\times {10}^{9}$ V/m. Moreover, the self-diffusion coefficients at ${E}_{{\rm{\max }}}=9.0\times {10}^{9}$ are even lower than in the case of ${E}_{{\rm{\max }}}=zero$, it seems that the strong field even hinders molecular diffusion. Figure 4 indicates that the application of microwave can reduce the diffusion coefficient, which is the direct evidence of the presence of microwave non-thermal effects. It is known that microwave fields can lead to molecular dipole moments continuous rotate to align the external electric field. Combining with the almost constancy of the first peak position shown in Fig. 2, thus, the reduction of self-diffusion coefficient was attributed to the molecular rearrangement in fluid structuring.

### Hydrogen bonding dynamics

The hydrogen bonding dynamics are detected to further analyse the effects of hydrogen bonds on the variation of self-diffusion coefficient in DMSO-ethanol mixtures under microwave field. Here, hydrogen bonds are defined by the following geometric criteria: ${r}_{OO} < 0.35\,nm$ and ${\varphi }_{OO} < 30^\circ$, where *r*_*OO*_ is the distance between the donor and acceptor oxygen atoms and *ϕ*_*OO*_ is the angle between the intramolecular O−H bond and *r*_*OO*_^67^.

**Figure 5:**
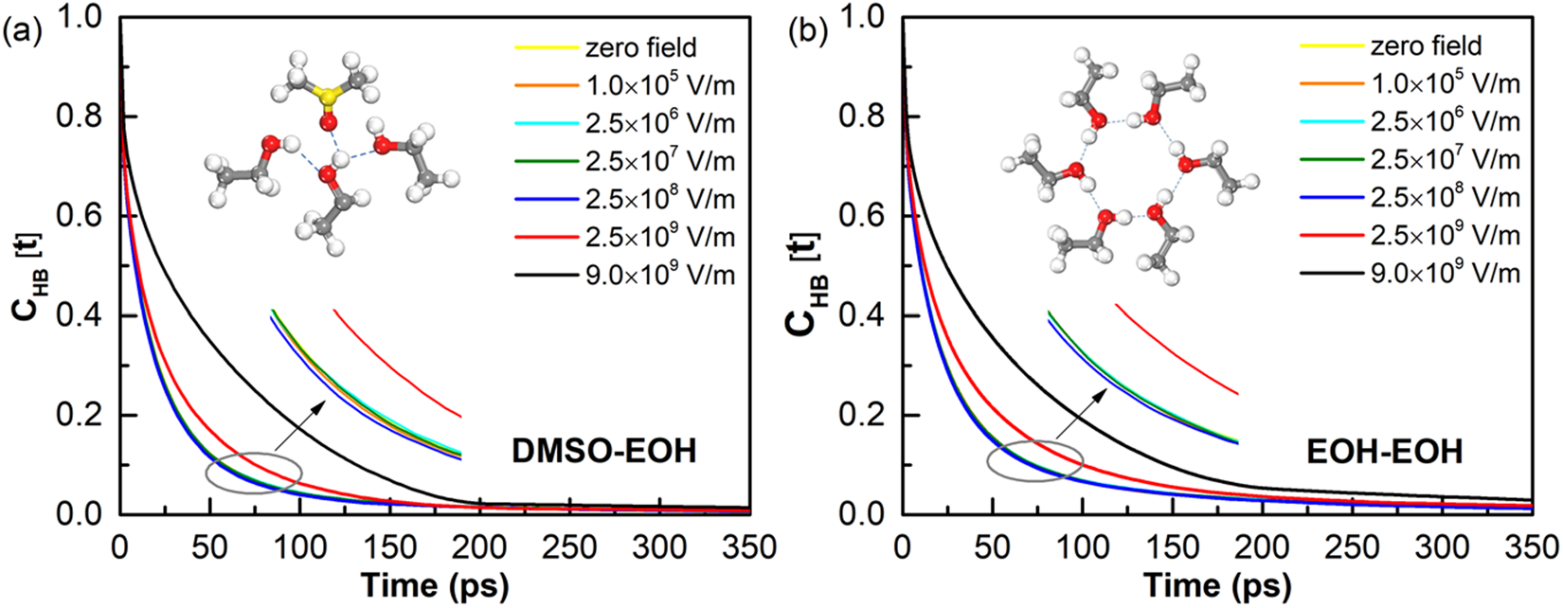
Hydrogen bonding autocorrelation functions in DMSO-ethanol mixtures versus simulation time with different field intensities: (**a**) DMSO-ethanol; (**b**) ethanol-ethanol. Where the curves represented the field intensity below $2.5\times {10}^{7}$ V/m are completely coincides with the curve represented the ${E}_{{\rm{\max }}}=2.5\times {10}^{8}$ V/m.

Assume that the hydrogen bond is intact at time zero with the bonding state, the probability that it was intact at time *t* can be described by the autocorrelation function *C*_*HB*_(*t*)^68^. The dynamics of *C*_*HB*_(*t*) evaluating the hydrogen-bond structural relaxation and the associated relaxation time can be interpreted as the time-scale reorganization of hydrogen bonds^69–73^. The time-dependence *C*_*HB*_(*t*) under different strengths of microwave field are shown in Fig. 5. There is a threshold effect at about $2.5\times {10}^{8}$ V/m, when the field strength is bigger than this threshold value, the decay rate of *C*_*HB*_(*t*) decreases as strengthening the e/m field. The hydrogen bonding lifetimes is obtained from the long time decay of the autocorrelation function ${C}_{HB}(t)= < \,{\eta }_{ij}(t){\eta }_{ij}(0) > {\eta }_{ij}{(0)}^{2}\cong \exp (\,-\,t/\tau )$, with ${\eta }_{ij}(t)$ takes the values 0 or 1 depending on the hydrogen-bond state of a given pair of oxygen *i* and hydrogen *j* at time *t*^74^. The single-exponential relation between *C*_*HB*_(*t*) and *τ* is utilized to get the changing trend of the hydrogen bonding lifetimes with the microwave field rather than the accurate value. The corresponding hydrogen bonding lifetimes are listed in Table 1.

**Table 1:** The hydrogen bonding lifetimes of DMSO-ethanol and ethanol-ethanol.

	Zero field^b^	1E5^b^	2.5E6^b^	2.5E7^b^	2.5E8^b^	2.5E9^b^	9E9^b^
*τ*_*DE*_^a^	18.26	18.09	18.08	18.12	17.54	22.58	47.58
*τ*_*EE*_^a^	21.65	21.65	21.54	20.86	20.98	28.78	51.33

^a^The unit of hydrogen bonding lifetime is ps, and the error is ±5%.

^b^The unit of the intensity of microwave field is *V/m*.

As shown in Table 1, hydrogen bonding lifetimes are apparently prolonged at field intensities larger than 2.5 × 10^8^ V/m, which is coincident with the threshold effect mentioned previous paragraph, indicating that the hydrogen bonds between ethanol and DMSO are significantly strengthened with increasing the field strength. What’s more, hydrogen bonding lifetimes of DMSO-ethanol mixture are about three times longer than that of the pure water^75^, which also demonstrates the stronger hydrogen bond interaction in the mixture. It is well known that the faster diffusion will result in faster hydrogen bond relaxation and vice versa^69^. The mechanism by which the mobility distinctly decrease in Fig. 4 can ascribe the longer lifetimes of hydrogen bonds.

**Figure 6:**
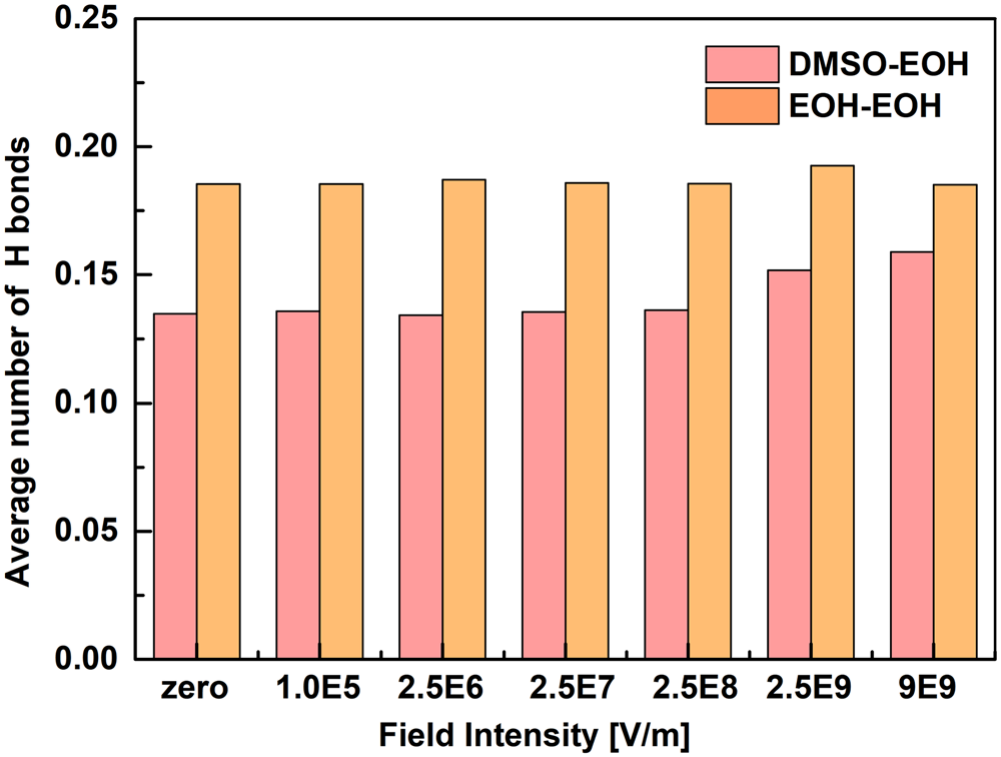
The average hydrogen bonding numbers of the DMSO-ethanol and the ethanol-ethanol as the function of field intensity.

The molecular rotation following the external electric field should affects hydrogen bonding, which is strongly dependent on molecular orientation between interacting pairs^76^; thus, the variation in the average number of hydrogen bonds with the implementation of the microwave was calculated (Fig. 6). The results in Fig. 6, the average hydrogen bonding numbers of ethanol-ethanol show minor changes, but a slightly increase for DMSO-ethanol in large field intensity, it is therefore logical that microwave fields boost molecular rotation and provides more opportunities for creating hydrogen bonds between DMSO and ethanol.

### Intermolecular interaction energy

**Figure 7:**
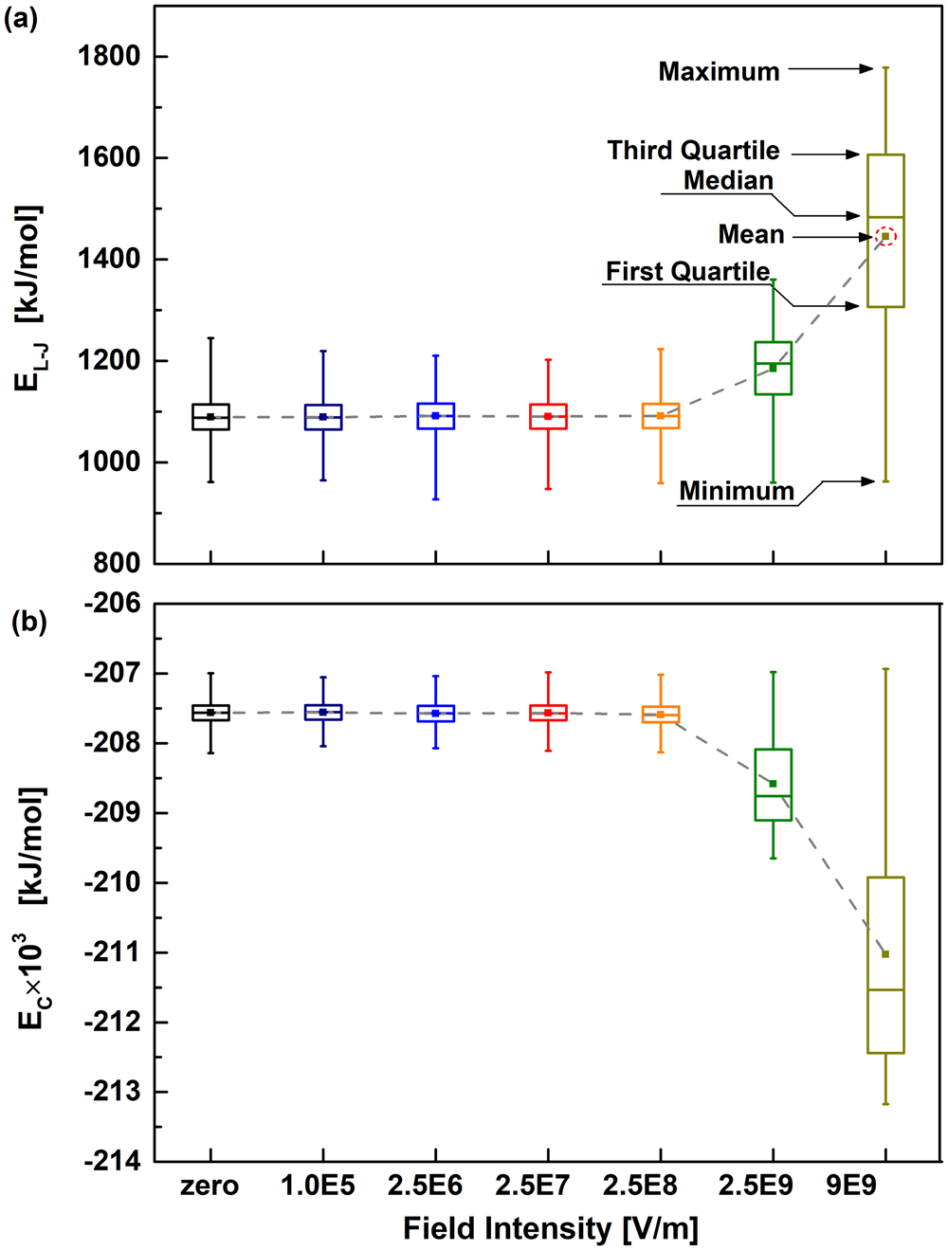
DMSO-ethanol intermolecular interaction energy, split in (**a**) Lennard-Jones and (**b**) Coulombic under microwave fields as a function of field intensity. The values are obtained by statistical analysis of time-dependence intermolecular energy extracted from a 5 ns equilibrium simulation. Dash lines are showed for guiding purposes.

In order to further interpret the properties changes mentioned above, intermolecular energy of DMSO-ethanol compound, split in their Lennard-Jones and Coulombic contributions, as a function of field intensity is reported in Fig. 7. As can be seen from the diagram, increasing field intensities lead to increase average interaction energy, in absolute value. This behavior of intermolecular energy would justify the lower diffusivities and longer hydrogen bonding lifetimes displayed in Figs 4 and 6. Moreover, from the two quartiles and median lines in boxplot, both instantaneous Lennard-Jones and Coulombic potential energy show strong vibration when the applied field intensities are larger than 2.5 × 10^8^ V/m. Nevertheless, the intermolecular energy is almost no change at all for the weak fields, intensities lower than 2.5 × 10^8^ V/m, which is accordance with the “critical point” in experiment. Moreover, Fig. 7 also illustrates that microwave energy was redistributed and partially stored as the intermolecular interaction potential energy, leading to significantly strengthen of the hydrogen bonding network.

## Conclusions

In this paper, molecular dynamics simulation of DMSO-ethanol mixture were performed under microwave fields ranging from 0 to 9.0 × 10^9^ V/m to investigate the nonlinear characteristic of output-input microwave power emerged in experiment. The effects of microwave field on the structure, transport property, hydrogen bonding dynamics and intermolecular interaction energy were analyzed. These properties show a pronounced threshold effect (about ${E}_{{\rm{\max }}}=2.5\times {10}^{8}$), which is consistent with the experiment. Stronger fields have little effect on the conformation of the mixture but lead to a remarked decrease in molecular diffusion. The increased intermolecular interaction energy, arising from redistribution of microwave energy, alters the hydrogen bonding arrangement dynamics, and prolongs lifetimes of hydrogen bond as a result. The tangible effects of microwave on DMSO-ethanol mixture were estimated in MD simulation via the sufficiently intense e/m field, which is expected to further interpret the mechanism of non-thermal effect.

## Methods

### Interaction Potentials

In all simulations, the OPLS-AA^77–79^ model was used for both DMSO and ethanol molecules. The nonbonded interactions are given by a sum of Lennard-Jones and Coulomb terms^80^,

2 $${E}_{ab}=\sum _{i}^{ona}\sum _{j}^{onb}[{q}_{i}{q}_{j}{e}^{2}/{r}_{ij}+4{\varepsilon }_{ij}({{\sigma }_{ij}}^{12}/{{r}_{ij}}^{12}-{{\sigma }_{ij}}^{6}/{{r}_{ij}}^{6})]\,{f}_{ij}$$

where *E*_*ab*_ is the interaction energy between molecules a and b. The Lennard-Jones interaction parameters (*ε*_*ij*_ and *σ*_*ij*_) between sites *i* and *j* on distinct molecules are set by combining rules, ${\varepsilon }_{ij}=\sqrt{({\varepsilon }_{ii}{\varepsilon }_{jj})}$ and ${\sigma }_{ij}=\sqrt{({\sigma }_{ii}{\sigma }_{jj})}$. *q*_*i*_ is the partial charge on site *i* and *r* is the separation between these sites. In this equation, *f*_*ij*_ is the correction factor for the Lennard-Jones 1–4 interaction, equal to 2. The potential parameters *q*_*i*_, *ε*_*ij*_, and *σ*_*ij*_ for DMSO and ethanol, and corresponding molecular structures were displayed in Supplementary Materials. (Table 1 and Scheme 1).

### Application of an External Electric Field

**Figure 8:**
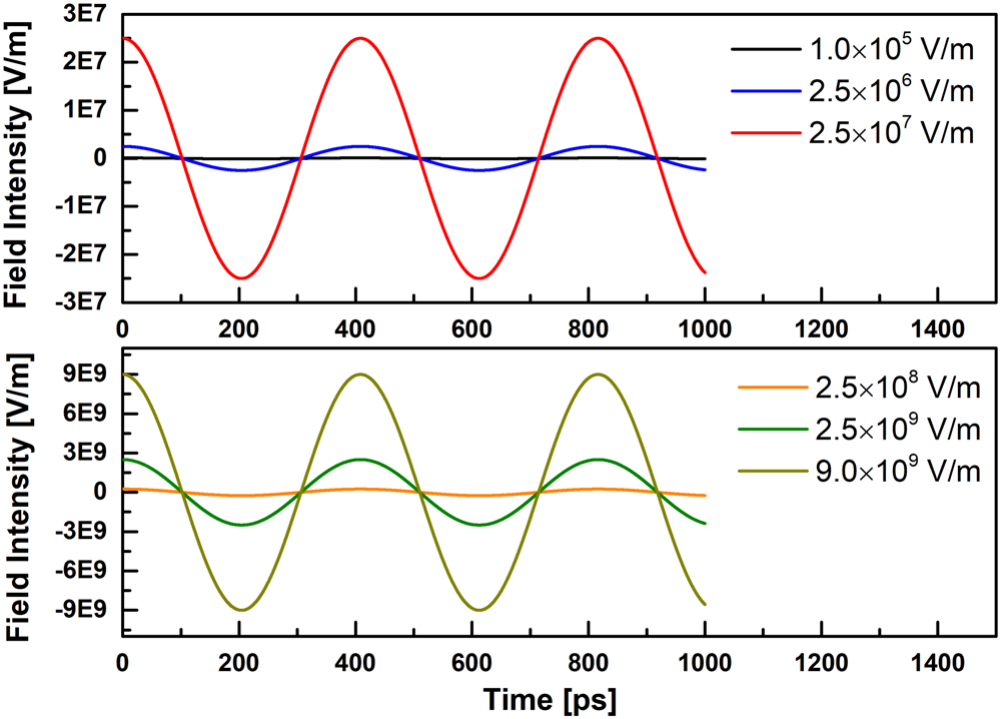
The amplitude of applied microwave field.

The homogeneous microwave is applied along the *x*-axis direction, and it is represented by spatially uniform, time-alternating electric field of the form

3 $$E(t)={E}_{{\rm{\max }}}\,\cos (\omega t)(1\overrightarrow{i}+0\overrightarrow{j}+0\overrightarrow{k}),\,B=0$$

where *E*_max_ and *ω* stands for the field amplitude and frequency, respectively. The applied external electric fields were of frequency $\omega =2.45$ GHz and of intensity ${E}_{{\rm{\max }}}=1.0\times {10}^{5}$, $2.5\times {10}^{6}$, $2.5\times {10}^{7}$, $2.5\times {10}^{8}$, $2.5\times {10}^{9}$, and $9.0\times {10}^{9}$ V/m, respectively (Fig. 8). Note that although the field’s intensities applied in the simulation are several order of magnitude larger than that in experiment ($1.0\times {10}^{5}$ V/m), in fact, due to the microwave attenuate in the dielectric medium, the microwave field surrounding vacuum space should be considerably lager compared with the actual microwave field within the experimental sample^81^. Furthermore, it has been proposed that applying e/m field intensity of the order of 0.1 V/Å is necessary to observe tangible effects within limited nanosecond time scales^82^. Thus, strengths of e/m field applied in this simulation are reasonable.

### Simulation Details

All simulations were carried out using the GROMACS 4.6.3^83^ simulation package. Initial simulation systems were built using the Packmol program, including 420 ethanol molecules and 604 DMSO molecules in a cubic box with a length of 4.5 nm. Periodic boundary conditions were imposed in all three dimensions. Real-space Ewald interactions and van der Waals interactions were truncated at 10 Å. Particle Mesh-Ewald method^77,78^ was applied to handle long-range electrostatics to within a relative tolerance of $1.0\times {10}^{-6}$. And the LINCS algorithm^79^ was applied to constrain bond lengths of hydrogen atoms. Prior to system relaxation MD, the energy of the initial configurations was performed with a protocol of steepest descent, using termination gradients of 5000 *kJ*/*mol*·*nm*. And it is prior to production simulations, the systems were simulated on the canonical (*N*, *V*, *T*) ensemble for 1 *ns* at 300* K*, using velocity-rescaling thermostat^84^ to maintain the system temperature in order to study the non-thermal effects of the electromagnetic field on the DMSO-ethanol mixtures, and isotropic (*N*, *P*, *T*) ensemble for 1 *ns* at 1 *bar*, using Parrinello-Rahman barostat^85,86^. The external fields were applied in conjunction with NPT coupling, which are referred as nonequilibrium NNPT simulation, in order to isolate athermal effects as much as possible from thermal effects. A series of NNPT simulation was carried out for 5 ns, as well as equilibrium, zero-fields simulations. A leapfrog algorithm^87^ was accompanied by a time step of 2 *fs*.

## Electronic supplementary material

Supplementary Information

## References

[1] de la Hoz, A., Díaz-Ortiz, A. & Prieto, P. Microwave-Assisted Green Organic Synthesis (2016).

[2] Sharma, N., Sharma, U. & Van der Eycken, E. Microwave Assisted Organic Synthesis: Overview of Recent applicatioins (2016).

[3] Frecentesec F (2016). Microwave Assisted Organic Synthesis of Heterocycles in Aqueous Media: Recent Advances in Medicinal Chemistry. Medicinal Chemistry.

[4] Leadbeater, N. 9.10 Organic Synthesis Using Microwave Heating (2014).

[5] Kappe CO (2004). Controlled microwave heating in modern organic synthesis. Angewandte Chemie International Edition.

[6] Bogdal, D. & Prociak, A. *Microwave-enhanced polymer chemistry and technology*. (John Wiley & Sons, 2008).

[7] Wiesbrock, F., Hoogenboom, R. & Schubert, U. S. (SPRINGER-VERLAG BERLIN HEIDELBERGER PLATZ 3, D-14197 BERLIN, GERMANY, 2016).

[8] Tsuji M, Hashimoto M, Nishizawa Y, Kubokawa M, Tsuji T (2005). Microwave‐assisted synthesis of metallic nanostructures in solution. Chemistry-A European Journal.

[9] Horikoshi, S. & Serpone, N. *Microwaves in nanoparticle synthesis: fundamentals and applications*. (John Wiley & Sons, 2013).

[10] Bilecka I, Niederberger M (2010). Microwave chemistry for inorganic nanomaterials synthesis. Nanoscale.

[11] Rahman, K. M. & Thurston, D. E. Effect of microwave irradiation on covalent ligand–DNA interactions. *Chemical Communications*, 2875–2877 (2009). 10.1039/b902357g 19436895

[12] Damm M (2012). Can electromagnetic fields influence the structure and enzymatic digest of proteins? A critical evaluation of microwave-assisted proteomics protocols. Journal of proteomics.

[13] Yoshimura, T., Mineki, S. & Ohuchi, S. Microwave-Assisted Enzymatic Reactions. *Microwaves in nanoparticle synthesis: Fundamentals and applications* (2015).

[14] Horikoshi S, Nakamura K, Kawaguchi M, Kondo J, Serpone N (2016). Effect of microwave radiation on the activity of catalase. decomposition of hydrogen peroxide under microwave and conventional heating. RSC Advances.

[15] Kappe CO, Dallinger D (2009). Controlled microwave heating in modern organic synthesis: highlights from the 2004–2008 literature. Molecular diversity.

[16] Loupy, A. Microwaves in organic synthesis. 2nd edn, (Wiley-Vch Weinheim, 2006).

[17] Collins JM, Leadbeater NE (2007). Microwave energy: a versatile tool for the biosciences. Organic & biomolecular chemistry.

[18] Zhou, J. *et al*. A new type of power energy for accelerating chemical reactions: the nature of a microwave-driving force for accelerating chemical reactions. *Scientific reports* **6** (2016). 10.1038/srep25149 PMC4846869 27118640

[19] Kappe CO, Pieber B, Dallinger D (2013). Microwave effects in organic synthesis: myth or reality?. Angewandte Chemie International Edition.

[20] Bhattacharjee MK, Delsol JK (2014). Does microwave sterilization of growth media involve any non-thermal effect?. Journal of microbiological methods.

[21] Huang K, Yang X, Hua W, Jia G, Yang L (2009). Experimental evidence of a microwave non-thermal effect in electrolyte aqueous solutions. New Journal of Chemistry.

[22] de la Hoz A, Diaz-Ortiz A, Moreno A (2005). Microwaves in organic synthesis. Thermal and non-thermal microwave effects. Chemical Society Reviews.

[23] Herrero MA, Kremsner JM, Kappe CO (2008). Nonthermal microwave effects revisited: on the importance of internal temperature monitoring and agitation in microwave chemistry. The Journal of organic chemistry.

[24] Wang J (2006). Evidence for the microwave effect during hybrid sintering. Journal of the American Ceramic Society.

[25] Whittaker A (2005). Diffusion in microwave-heated ceramics. Chemistry of Materials.

[26] de la Hoz A, Díaz-Ortiz A, Moreno A (2006). Review on non-thermal effects of microwave irradiation in organic synthesis. Journal of Microwave Power and Electromagnetic Energy.

[27] Porcelli M (1997). Non‐thermal effects of microwaves on proteins: thermophilic enzymes as model system. FEBS letters.

[28] Tsujita, K. Y., Yoshimizu, C. H. & Miyamoto, C. M. U.S. Patent No. US6706088 B2 (2004).

[29] Nakai Y, Tsujita Y, Yoshimizu H (2002). Control of gas permeability for cellulose acetate membrane by microwave irradiation. Desalination.

[30] Gopalakrishnan S, Münch J, Herrmann R, Schwieger W (2006). Effects of microwave radiation on one-step oxidation of benzene to phenol with nitrous oxide over Fe-ZSM-5 catalyst. Chemical Engineering Journal.

[31] Kataoka, D. T. *et al*. Proceedings of the Second World Congress on Microwave and Radio Frequency Processing, Orlando, FL, 2–6 April 2000 American Ceramic Society (2000).

[32] Al-Obeidi F, Austin RE, Okonya JF, Bond DR (2003). Microwave-assisted solid-phase synthesis (MASS): parallel and combinatorial chemical library synthesis. Mini reviews in medicinal chemistry.

[33] Tye H, Whittaker M (2004). Use of a Design of Experiments approach for the optimisation of a microwave assisted Ugi reaction. Organic & biomolecular chemistry.

[34] Harriman GC (1997). Synthesis of small and medium sized 2, 2-disubstituted lactams via the “intramolecular” three component Ugi reaction. Tetrahedron letters.

[35] Purdue MJ, MacElroy J, O’Shea D, Okuom MO, Blum FD (2006). A comparative study of the properties of polar and nonpolar solvent/solute/polystyrene solutions in microwave fields via molecular dynamics. The Journal of chemical physics.

[36] Mayo KG, Nearhoof EH, Kiddle JJ (2002). Microwave-accelerated ruthenium-catalyzed olefin metathesis. Organic letters.

[37] Huang KM, Jia GZ, Yang XQ (2008). Nonlinear characteristics of conductivity in aqueous NaCl solution at microwave frequency. Acta Physico-Chimica Sinica.

[38] Saitta AM, Saija F, Giaquinta PV (2012). Ab initio molecular dynamics study of dissociation of water under an electric field. Physical review letters.

[39] Shneider M, Pekker M (2013). Non-thermal mechanism of weak microwave fields influence on neurons. Journal of applied physics.

[40] Horikoshi S, Watanabe T, Kamata M, Suzuki Y, Serpone N (2015). Microwave-assisted organic syntheses: microwave effect on intramolecular reactions–the Claisen rearrangement of allylphenyl ether and 1-allyloxy-4-methoxybenzene. RSC Advances.

[41] Ahirwar R, Tanwar S, Bora U, Nahar P (2016). Microwave non-thermal effect reduces ELISA timing to less than 5 minutes. RSC Advances.

[42] Loupy A, Maurel F, Sabatié-Gogová A (2004). Improvements in Diels–Alder cycloadditions with some acetylenic compounds under solvent-free microwave-assisted conditions: experimental results and theoretical approaches. Tetrahedron.

[43] Zhang Y-M (2014). Research on epoxy resin decomposition under microwave heating by using ReaxFF molecular dynamics simulations. RSC Advances.

[44] Tian W-Y, Huang K-M, Yang L-J, Guo Y-N, Liu F-H (2014). Investigate the microscopic properties and the non-thermal effect of the electrolyte solution under microwave irradiation. Chemical Physics Letters.

[45] Floros S, Liakopoulou-Kyriakides M, Karatasos K, Papadopoulos GE (2017). Frequency Dependent Non-Thermal Effects of Oscillating Electric Fields in the Microwave Region on the Properties of a Solvated Lysozyme System: A Molecular Dynamics Study. PloS one.

[46] Sun H, Huang K (2015). Experimental study of dielectric property changes in DMSO–primary alcohol mixtures under low-intensity microwaves. Rsc Advances.

[47] Wu XF, Natte K (2016). The Applications of Dimethyl Sulfoxide as Reagent in Organic Synthesis. Advanced Synthesis & Catalysis.

[48] Vitkovskaya, N. M. *et al*. Exploring acetylene chemistry in superbasic media: A theoretical study of the effect of water on vinylation and ethynylation reactions with acetylene in KOH/DMSO and NaOH/DMSO systems. *Journal of Physical Organic Chemistry* **30** (2017).

[49] Attarian, S., Janakiram, M., Ezzati, A. & Gucalp, R. A. (American Society of Clinical Oncology, 2017).

[50] Majdi S, Najafinobar N, Dunevall J, Lovric J, Ewing AG (2017). DMSO Chemically Alters Cell Membranes to Slow Exocytosis and Increase the Fraction of Partial Transmitter Released. ChemBioChem.

[51] Roy S, Banerjee S, Biyani N, Jana B, Bagchi B (2010). Theoretical and computational analysis of static and dynamic anomalies in water− DMSO binary mixture at low DMSO concentrations. The Journal of Physical Chemistry B.

[52] Lu Z, Manias E, Macdonald DD, Lanagan M (2009). Dielectric relaxation in dimethyl sulfoxide/water mixtures studied by microwave dielectric relaxation spectroscopy. The Journal of Physical Chemistry A.

[53] Gereben O, Pusztai Ls (2015). Investigation of the structure of ethanol–water mixtures by molecular dynamics simulation I: analyses concerning the hydrogen-bonded pairs. The Journal of Physical Chemistry B.

[54] Benmore CJ, Loh YL (2000). The structure of liquid ethanol: A neutron diffraction and molecular dynamics study. The Journal of Chemical Physics.

[55] Dhumal NR (2011). Electronic structure, molecular electrostatic potential and hydrogen bonding in DMSO–X complexes (X = ethanol, methanol and water). Spectrochimica Acta Part A: Molecular and Biomolecular Spectroscopy.

[56] Noack K, Kiefer J, Leipertz A (2010). Concentration‐Dependent Hydrogen‐Bonding Effects on the Dimethyl Sulfoxide Vibrational Structure in the Presence of Water, Methanol, and Ethanol. ChemPhysChem.

[57] Guo-Zhu J, Jie Q (2014). Dielectric constant of dimethyl sulfoxide-monohydric alcohol mixture solution at the microwave frequency. Fluid Phase Equilibria.

[58] Jie Q, Guo-Zhu J (2013). Dielectric Constant of Polyhydric Alcohol–DMSO Mixture Solution at the Microwave Frequency. The Journal of Physical Chemistry A.

[59] English NJ, MacElroy J (2003). Molecular dynamics simulations of microwave heating of water. The Journal of chemical physics.

[60] English NJ, MacElroy J (2003). Hydrogen bonding and molecular mobility in liquid water in external electromagnetic fields. The Journal of chemical physics.

[61] English N (2006). Molecular dynamics simulations of microwave effects on water using different long-range electrostatics methodologies. Molecular Physics.

[62] English NJ, MacElroy J (2004). Theoretical studies of the kinetics of methane hydrate crystallization in external electromagnetic fields. The Journal of chemical physics.

[63] English NJ, Sorescu DC, Johnson JK (2006). Effects of an external electromagnetic field on rutile Tio 2: A molecular dynamics study. Journal of Physics and Chemistry of Solids.

[64] Blanco C, Auerbach SM (2002). Microwave-Driven Zeolite− Guest Systems Show Athermal Effects from Nonequilibrium Molecular Dynamics. Journal of the American Chemical Society.

[65] Blanco C, Auerbach SM (2003). Nonequilibrium Molecular Dynamics of Microwave-Driven Zeolite− Guest Systems: Loading Dependence of Athermal Effects. The Journal of Physical Chemistry B.

[66] Yang L, Huang K (2010). Electric conductivity in electrolyte solution under external electromagnetic field by nonequilibrium molecular dynamics simulation. The Journal of Physical Chemistry B.

[67] Luzar A, Chandler D (1993). Structure and hydrogen bond dynamics of water–dimethyl sulfoxide mixtures by computer simulations. The Journal of chemical physics.

[68] Sun W, Chen Z, Huang S-Y (2005). Molecular dynamics simulation of liquid methanol under the influence of an external electric field. Fluid phase equilibria.

[69] Chanda J, Chakraborty S, Bandyopadhyay S (2006). Sensitivity of hydrogen bond lifetime dynamics to the presence of ethanol at the interface of a phospholipid bilayer. The Journal of Physical Chemistry B.

[70] Guardia E, Marti J, Padro J, Saiz L, Komolkin A (2002). Dynamics in hydrogen bonded liquids: water and alcohols. Journal of molecular liquids.

[71] Elola MD, Ladanyi BM (2006). Computational study of structural and dynamical properties of formamide-water mixtures. The Journal of chemical physics.

[72] Lee H-S, Tuckerman ME (2007). Dynamical properties of liquid water from ab initio molecular dynamics performed in the complete basis set limit. The Journal of chemical physics.

[73] Chen C, Li WZ, Song YC, Yang J (2009). Hydrogen bonding analysis of glycerol aqueous solutions: a molecular dynamics simulation study. Journal of Molecular Liquids.

[74] van der Spoel D, van Maaren PJ, Larsson P, Tîmneanu N (2006). Thermodynamics of hydrogen bonding in hydrophilic and hydrophobic media. The Journal of Physical Chemistry B.

[75] Zhu H, Ghoufi A, Szymczyk A, Balannec B, Morineau D (2012). Anomalous dielectric behavior of nanoconfined electrolytic solutions. Physical review letters.

[76] Atilhan M, Aparicio S (2016). Behavior of Deep Eutectic Solvents under External Electric Fields: A Molecular Dynamics Approach. The Journal of Physical Chemistry B.

[77] Jorgensen WL, Tirado-Rives J (2005). Potential energy functions for atomic-level simulations of water and organic and biomolecular systems. Proceedings of the National Academy of Sciences of the United States of America.

[78] Dodda LS, Vilseck JZ, Tirado-Rives J, Jorgensen WL (2017). 1.14* CM1A-LBCC: Localized Bond-Charge Corrected CM1A Charges for Condensed-Phase Simulations. The Journal of Physical Chemistry B.

[79] Dodda, L. S., Cabeza de Vaca, I., Tirado-Rives, J. & Jorgensen, W. L. LigParGen web server: an automatic OPLS-AA parameter generator for organic ligands. Nucleic Acids Research (2017). 10.1093/nar/gkx312 PMC5793816 28444340

[80] Lei Y, Li H, Pan H, Han S (2003). Structures and hydrogen bonding analysis of N, N-dimethylformamide and N, N-dimethylformamide− water mixtures by molecular dynamics simulations. The Journal of Physical Chemistry A.

[81] Tanaka M, Sato M (2007). Microwave heating of water, ice, and saline solution: Molecular dynamics study. The Journal of chemical physics.

[82] English NJ, Solomentsev GY, O’Brien P (2009). Nonequilibrium molecular dynamics study of electric and low-frequency microwave fields on hen egg white lysozyme. The Journal of chemical physics.

[83] Hess B, Kutzner C, Van Der Spoel D, Lindahl E (2008). GROMACS 4: algorithms for highly efficient, load-balanced, and scalable molecular simulation. Journal of chemical theory and computation.

[84] Bussi G, Donadio D, Parrinello M (2007). Canonical sampling through velocity rescaling. The Journal of chemical physics.

[85] Parrinello M, Rahman A (1981). Polymorphic transitions in single crystals: A new molecular dynamics method. Journal of Applied physics.

[86] Nosé S, Klein M (1983). Constant pressure molecular dynamics for molecular systems. Molecular Physics.

[87] Van Gunsteren WF, Berendsen H (1988). A leap-frog algorithm for stochastic dynamics. Molecular Simulation.

