# Supplementary material for: Investigation of Nonlinear Output-Input Microwave Power of DMSO-Ethanol Mixture by Molecular Dynamics Simulation
Source: Sci Rep. 2018 May 8;8:7186. doi: 10.1038/s41598-018-21846-4 (PMC5940686; doi:10.1038/s41598-018-21846-4)
Supplement: Supplementary file 1 — Supplementary Information [file 41598_2018_21846_MOESM1_ESM.doc]

**Investigation of Nonlinear Output-Input Microwave Power of DMSO-Ethanol Mixture by Molecular Dynamics Simulation**

Min Zhou^1^, Ke Cheng^2^, Haoran Sun^2^, and Guozhu Jia^1^,*

*^1^College of Physical and Electronics Engineering, Sichuan Normal University, Chengdu, 610101, China*

*^2^College of Optoelectronic Technology, Chengdu University of Information Technology, Chengdu, 610103, China*

*Corresponding author. E-mail: [jia1689500@126.com](mailto:jia1689500@126.com)

**Supplementary Material**

Table 1. Potential parameters of the models employed.

atom m[g/mol] $\sigma$[Å] $\varepsilon$[kJ/mol] $q(e)$

Dimethyl sulfoxide (DMSO):

S 32.0600 0.3560 0.1653 1.3203

O 15.9990 0.2960 0.7113 -0.5616

C_1_(S-CH_3_) 12.0110 0.3500 0.2761 -0.8597

C_2_(S-CH_3_) 12.0110 0.3500 0.2761 -0.8590

H_1_(S-CH_3_) 1.0080 0.2500 0.1255 0.1598

H_2_(S-CH_3_) 1.0080 0.2500 0.1255 0.1602

Ethanol (EOH):

O 15.9990 0.3120 0.7113 -0.5957

C_1_(CH_2_) 12.0110 0.3500 0.2761 0.0142

C_2_(CH_3_) 12.0110 0.3500 0.2761 -0.2471

H(O-H) 1.0080 0.0000 0.0000 0.4103

H_1_(CH_2_) 1.0080 0.2500 0.1255 0.0646

H2(CH3) 1.0080 0.2500 0.1255 0.0964

Scheme 1. Structures of DMSO, and ethanol molecules^a^.





^a^Numbers was used to discriminate different types of carbon and hydrogen atoms.

Table 2. The first peak positions of atom-atom distribution functions for DMSO-ethanol mixtures as the function of field strength at 0.41 mole fraction of ethanol .

| g(r)  E(V/m) | O_D_-O_E_ | S_D_-O_E_ | O_D_-H_E_ | S_D_-H_E_ | O_D_-C_E_ | S_D_-C_E_ | O_E_-H_E_ | H_E_-H_E_ |
| --- | --- | --- | --- | --- | --- | --- | --- | --- |
| 0.0 | 0.272 | 0.312 | 0.18 | 0.326 | 0.356 | 0.45 | 0.18 | 0.254 |
| 1.0×10^5^ | 0.274 | 0.312 | 0.18 | 0.324 | 0.356 | 0.456 | 0.18 | 0.256 |
| 2.5×10^6^ | 0.274 | 0.312 | 0.18 | 0.33 | 0.352 | 0.46 | 0.18 | 0.254 |
| 2.5×10^7^ | 0.274 | 0.312 | 0.182 | 0.324 | 0.354 | 0.454 | 0.182 | 0.254 |
| 2.5×10^8^ | 0.272 | 0.314 | 0.182 | 0.324 | 0.354 | 0.456 | 0.182 | 0.254 |
| 2.5×10^9^ | 0.274 | 0.312 | 0.182 | 0.326 | 0.356 | 0.454 | 0.182 | 0.256 |
| 9.0×10^9^ | 0.274 | 0.312 | 0.18 | 0.326 | 0.356 | 0.456 | 0.18 | 0.252 |

Table 3. The first peak heights of atom-atom distribution functions for DMSO-ethanol mixtures as the function of field strength at 0.41 mole fraction of ethanol .

| g(r)  E(V/m) | O_D_-O_E_ | S_D_-O_E_ | O_D_-H_E_ | S_D_-H_E_ | O_D_-C_E_ | S_D_-C_E_ | O_E_-H_E_ | H_E_-H_E_ |
| --- | --- | --- | --- | --- | --- | --- | --- | --- |
| 0.0 | 1.99324 | 2.33783 | 3.81508 | 1.65733 | 1.12136 | 1.15372 | 3.81508 | 3.64061 |
| 1.0×10^5^ | 1.99728 | 2.33934 | 3.80169 | 1.65574 | 1.12498 | 1.16102 | 3.80169 | 3.62637 |
| 2.5×10^6^ | 1.97112 | 2.32623 | 3.75029 | 1.6375 | 1.10896 | 1.15109 | 3.75029 | 3.64316 |
| 2.5×10^7^ | 1.99865 | 2.33717 | 3.85526 | 1.65161 | 1.12458 | 1.15586 | 3.85526 | 3.56684 |
| 2.5×10^8^ | 1.99513 | 2.35709 | 3.79315 | 1.65334 | 1.12673 | 1.15939 | 3.79315 | 3.65069 |
| 2.5×10^9^ | 1.91788 | 2.38681 | 3.66026 | 1.59603 | 1.19365 | 1.1892 | 3.66026 | 3.59741 |
| 9.0×10^9^ | 1.83843 | 2.39595 | 3.53884 | 1.54557 | 1.35862 | 1.29805 | 3.53884 | 3.22435 |

Table 4. The second peak positions of atom-atom distribution functions for DMSO-ethanol mixtures as the function of field strength at 0.41 mole fraction of ethanol .

| g(r)  E(V/m) | O_D_-O_E_ | S_D_-O_E_ | O_D_-H_E_ | S_D_-H_E_ | O_D_-C_E_ | S_D_-C_E_ | O_E_-H_E_ | H_E_-H_E_ |
| --- | --- | --- | --- | --- | --- | --- | --- | --- |
| 0.0 | 0.366 | 0.404 | 0.464 | 0.764 | 0.48 | 0.532 | 0.344 | 0.508 |
| 1.0×10^5^ | 0.364 | 0.404 | 0.462 | 0.772 | 0.478 | 0.532 | 0.344 | 0.508 |
| 2.5×10^6^ | 0.368 | 0.404 | 0.462 | 0.752 | 0.478 | 0.53 | 0.342 | 0.51 |
| 2.5×10^7^ | 0.372 | 0.404 | 0.462 | 0.758 | 0.48 | 0.536 | 0.346 | 0.51 |
| 2.5×10^8^ | 0.378 | 0.404 | 0.464 | 0.748 | 0.482 | 0.538 | 0.348 | 0.512 |
| 2.5×10^9^ | 0.362 | 0.404 | 0.458 | 0.752 | 0.482 | 0.53 | 0.344 | 0.51 |
| 9.0×10^9^ | 0.362 | 0.408 | 0.47 | 0.732 | 0.482 | 0.526 | 0.338 | 0.512 |

Table 5. The second peak heights of atom-atom distribution functions for DMSO-ethanol mixtures as the function of field strength at 0.41 mole fraction of ethanol .

| g(r)  E(V/m) | O_D_-O_E_ | S_D_-O_E_ | O_D_-H_E_ | S_D_-H_E_ | O_D_-C_E_ | S_D_-C_E_ | O_E_-H_E_ | H_E_-H_E_ |
| --- | --- | --- | --- | --- | --- | --- | --- | --- |
| 0.0 | 0.98776 | 1.08814 | 1.06127 | 1.06781 | 0.96357 | 1.45536 | 1.31343 | 0.85442 |
| 1.0×10^5^ | 0.99265 | 1.08837 | 1.06136 | 1.06671 | 0.96724 | 1.45974 | 1.31839 | 0.85974 |
| 2.5×10^6^ | 0.98142 | 1.08482 | 1.0614 | 1.06467 | 0.95886 | 1.45029 | 1.33485 | 0.85625 |
| 2.5×10^7^ | 0.98625 | 1.099 | 1.07115 | 1.07076 | 0.96555 | 1.45597 | 1.31321 | 0.86311 |
| 2.5×10^8^ | 0.99167 | 1.09006 | 1.06996 | 1.06951 | 0.97213 | 1.45824 | 1.32563 | 0.85813 |
| 2.5×10^9^ | 1.03143 | 1.11187 | 1.09458 | 1.09094 | 0.9683 | 1.54598 | 1.32257 | 0.88864 |
| 9.0×10^9^ | 1.00398 | 1.10121 | 1.08888 | 1.09957 | 0.94239 | 1.62469 | 1.19874 | 0.9554 |
